# Whole-Genome Sequence of Bacillus megaterium Strain SGAir0080, Isolated from an Indoor Air Sample

**DOI:** 10.1128/MRA.01249-19

**Published:** 2019-12-12

**Authors:** Namrata Kalsi, Akira Uchida, Rikky W. Purbojati, James N. I. Houghton, Caroline Chénard, Anthony Wong, Megan E. Clare, Kavita K. Kushwaha, Alexander Putra, Nicolas E. Gaultier, Balakrishnan N. V. Premkrishnan, Cassie E. Heinle, Vineeth Kodengil Vettath, Daniela I. Drautz-Moses, Ana Carolina M. Junqueira, Stephan C. Schuster

**Affiliations:** a Singapore Centre for Environmental Life Sciences Engineering, Nanyang Technological University, Singapore; b Departamento de Genética, Instituto de Biologia, Universidade Federal do Rio de Janeiro, Rio de Janeiro, Brazil; University of Rochester School of Medicine and Dentistry

## Abstract

Bacillus megaterium strain SGAir0080 was isolated from a tropical air sample in Singapore. Its genome was assembled using single-molecule real-time (SMRT) sequencing and MiSeq reads. It has one chromosome of 5.06 Mbp and seven plasmids (average length, 62.8 kbp). It possesses 5,339 protein-coding genes, 130 tRNAs, and 35 rRNAs.

## ANNOUNCEMENT

Bacillus megaterium (*Firmicutes*) is an aerobic, spore-forming, Gram-positive species (1) that was first described by De Bary in 1884 (2). In the field of biotechnology, B. megaterium is used in the production of intracellular recombinant proteins (3–6). Although B. megaterium is found predominantly in soil (7), it is ubiquitous in the environment and has been reported from air samples (8).

B. megaterium was sequenced here in an effort to investigate the diverse microbiological communities present in air. The B. megaterium strain SGAir0080 was isolated in Singapore (global positioning system [GPS] coordinates 1.347654 N, 103.685240 E) from an air sample collected using the Andersen single-stage impactor (SKC, Inc., USA) and impacted onto marine agar (Becton, Dickinson, USA). Colonies were isolated by growing cultures overnight on Trypticase soy agar (Becton, Dickinson) at 30°C, followed by cultivation overnight in lysogeny broth at 30°C prior to DNA extraction. DNA was purified using the Wizard genomic DNA purification kit (Promega, USA). A genomic DNA library was then prepared with the SMRTbell template prep kit 1.0 (Pacific Biosciences, USA). DNA was sheared using the g-Tube shearing method and size selected using BluePippin size selection (cutoff, 15 kb). This was followed by single-molecule real-time (SMRT) sequencing on a PacBio RS II sequencer (DNA sequencing kit 4.0 v2). Whole-genome shotgun libraries were constructed using the TruSeq Nano DNA library preparation kit (Illumina, USA), and short-read data were generated via a paired-end Illumina MiSeq run with a 300-bp read length. For the following analysis, all software was run with default settings unless otherwise stated.

Quality control of PacBio reads and MiSeq reads was performed using PreAssembler Filter v1 from the Hierarchical Genome Assembly Process v3 (HGAP3) (9) protocol, implemented in the PacBio SMRT Analysis 2.3.0 package and Cutadapt v1.8.1 (10), respectively. *De novo* assembly for 31,318 PacBio subreads (*N*_50,_ 16,423 bp) was performed using HGAP3 and polished with Quiver (9). The quality of the draft assembly was further improved with 697,928 MiSeq paired-end reads using Pilon v1.16 (11) (tracks –changes –vcf –fix all –mindepth 0.1 –mingap 10 –minmq 30 –minqual 20 –K 47). The complete assembly consists of 8 contigs (Table 1), with the chromosome having a G+C content of 38.2%. Contig lengths and G+C content were obtained with the Quality Assessment Tool for Genome Assemblies (QUAST) (12). Completeness and circularity of the chromosome and plasmids were evaluated using BUSCO (13) and Circlator v1.1.4 (14).

**TABLE 1:** Lengths and coverages of strain SGAir0080 contigs

Contig name	Length (bp)	Coverage (×)	GenBank accession no.
SGAir0080 chromosome	5,057,175	50.1	CP028084
SGAir0080 unnamed_1	122,231	39.1	CP028085
SGAir0080 unnamed_2	140,900	21.3	CP028088
SGAir0080 unnamed_3	37,586	16.4	CP028086
SGAir0080 unnamed_4	60,004	19.8	CP028087
SGAir0080 unnamed_6	24,808	13.8	CP028089
SGAir0080 unnamed_7	19,487	13.6	CP028090
SGAir0080 unnamed_8	35,203	17.6	CP028091

NCBI’s Prokaryotic Genome Annotation Pipeline (PGAP) v4.2 (15) was used for annotation, with a cutoff of 80% to determine the presence for a gene in the genome. This revealed 5,649 genes with 5,339 protein-coding genes, 35 rRNA genes (13 5S, 11 16S, and 11 23S), 130 tRNAs, 8 noncoding RNAs, and 137 pseudogenes.

Taxonomic identification was performed with Phyla-AMPHORA (16) using MarkerScanner.pl with an added “-DNA” flag and MarkerAlignTrim.pl with options “-WithReference” and “-OutputFormat phylip.” Phylotyping.pl was run with default parameters. SGAir0080 showed 99.3% identity with B. megaterium (minimum confidence, 1.0). Average nucleotide identity (ANI) analysis was performed with Microbial Species Identifier (MiSI) (17) against a database of 6,387 bacterial RefSeq genomes with a text filter for “type,synonym type, proxytype” and subsequent “getorf -find 3” option. This gave 97.5% similarity and an alignment fraction value of 0.73 to B. megaterium.

### Data availability.

The genome sequence of Bacillus megaterium strain SGAir0080 and its plasmids have been deposited in DDBJ/EMBL/GenBank under accession numbers CP028084, CP028085, CP028086, CP028087, CP028088, CP028089, CP028090, and CP028091, respectively, and in the SRA database under accession numbers SRR8894398 and SRR8894399, respectively.
